# The effectiveness of antibiotic cement-coated nails in post-traumatic femoral and tibial osteomyelitis – comparative analysis of custom-made versus commercially available nails

**DOI:** 10.5194/jbji-6-457-2021

**Published:** 2021-12-21

**Authors:** Germán Garabano, Hernán del Sel, Joaquin Anibal Rodriguez, Leonel Perez Alamino, Cesar Angel Pesciallo

**Affiliations:** Department of Orthopaedic and Traumatology, British Hospital of Buenos Aires, Perdriel 74 (C1280 AEB), Buenos Aires, Argentina

## Abstract

**Background**: The first objective of this retrospective study was to assess infection control rates in patients with chronic post-traumatic osteomyelitis (CPTO) of the femur or tibia treated with antibiotic cement-coated nails. The second objective was to compare the efficacy of custom-made nails versus commercially available antibiotic-coated nails in terms of infection control and need for reoperation. **Methods**: We reviewed a consecutive series of CPTO patients treated with antibiotic-coated nails who had a minimum follow-up of 24 months. We recorded the characteristics of the initial injury, the type of nail used, cement–nail debonding, infecting microorganisms, operating time, infection control, need for reoperation, and failure rate. We performed a comparative analysis between nails manufactured in the operating room (i.e., custom-made) and those commercially available. **Results**: Thirty patients were included. The affected bones were the femur ( n=15 ) and the tibia ( n=15 ). Twenty-one of the 30 initial injuries were open fractures. Staphylococcus aureus was the most frequently isolated microorganism (50 %). Sixteen patients were treated with custom-made nails and 14 with commercially available antibiotic-coated nails. At the time of extraction, four out of five custom-made antibiotic-coated nails experienced cement–bone debonding. Commercial nails were associated with shorter operating times ( p<0.0001 ). The overall infection control rate was 96.66 %. Eight (26.66 %) patients needed reoperation. There was one failure (3.33 %) in the group treated with custom-made antibiotic-coated nails. We did not find significant differences between nail types in terms of reoperation, infection control, and failure rate. **Conclusions**: The use of antibiotic cement-coated nails proved useful in CPTO treatment. Commercially available nails had significantly shorter operating times and did not present cement–bone debonding during removal. Our results seem to indicate that both nail types are similar in terms of infection control and reoperation rates.

## 1 Introduction

Post-traumatic osteomyelitis (PTO) is a challenging condition both for surgeons and patients (Hake et al., 2015). It can occur after the surgical treatment of closed or open fractures, though it is more frequent in the latter. Its reported prevalence ranges from 0.7 % to 33 % (Patzakis and Zalavras, 2005; Patzakis et al., 1986; Barger et al., 2017; Chadayammuri et al., 2017; Beals and Bryant, 2005).

At present, patient management practices include aggressive surgical debridement, bone stabilization, adequate soft tissue coverage, and the administration of specific antimicrobials depending on the infecting microorganism (Patzakis and Zalavras, 2005; Beals and Bryant, 2005; Tetsworth and Cierny, 1999; Cierny et al., 2003). Another important aspect to take into consideration is dead space management generated by the resection of devitalized tissue, bone sequestrum, and/or the removal of previous osteosynthesis (Hake et al., 2015; Tetsworth and Cierny, 1999; Cierny et al., 2003). Here host immune response and systemic antibiotic therapy tend to be weak (Cierny el al., 2003; Koury et al., 2017; Gosselin et al., 2004; Wasko and Borens, 2013). The use of antibiotic-loaded polymethylmethacrylate (PMMA) beads described by Klemm (1979) has proven effective for the local administration of antimicrobials; however, when the medullary cavity is affected by the infection – due to dissemination or colonization of an intramedullary nail – placing and removing the beads from this cavity is usually difficult (Klemm et al., 1998; Barger et al., 2017). In these cases, an antibiotic-coated intramedullary metal core can be used (Barger et al., 2017; Koury et al., 2017; Paley and Herzenberg, 2002; Bhadra and Roberts, 2009). Since they were first described, these devices have demonstrated efficacy in the treatment of long bone osteomyelitis with infection control rates of up to 80 %–100 % (Klemm et al., 1998; Bhadra and Roberts, 2009; Conway et al., 2014; Wasko and Kaminski, 2015; Makhdom et al., 2020).

Kanakaris et al. (2014) reported a 96 % success rate in a series of 24 PTO patients at 21 months of follow-up with the use of rods as a metal core.

Among other advantages, these intramedullary devices offer high local antibiotic concentration and may even provide mechanical support – depending on the type of metal core used in the manufacturing process (Barger et al., 2017; Kim et al., 2014).

While many studies in the literature have assessed the efficacy of antibiotic cement-coated nails (Wasko and Borens, 2013; Conway et al., 2014; Wasko and Kaminski, 2015; Kanakaris et al., 2014; Kim et al., 2014), few have made a comparative assessment of the results obtained with custom-made nails made in the operating room versus commercially available nails.

The main objective of this retrospective study was to assess the effectiveness of antibiotic cement-coated nails to control infections in a series of patients with chronic post-traumatic osteomyelitis (CPTO) of the femur or tibia. Our second objective was to compare infection control and reoperation and failure rates of custom-made versus commercially available antibiotic-coated nails.

## 2 Materials and methods

We retrospectively reviewed our department's database to identify consecutive patients treated with an antibiotic cement-coated nail for post-traumatic osteomyelitis between January 2010 and December 2017. The diagnosis of osteomyelitis was suspected when some of the following clinical, laboratory or imaging parameters were found: swelling, redness, pain, fever ( >38 ${}^{\circ }$ C), or active fistula or wound drainage with bone exposure or osteosynthesis; white blood cell count, erythrocyte sedimentation rate, and C-reactive protein; or cloacae, sequestra, involucrum, or periostitis on radiographs and/or MRI. Diagnosis was confirmed according to the Center for Disease Control and Prevention (CDC) guidelines, when at least one of the following criteria was present: growth of a microorganism in bone or soft tissue culture of intraoperative samples, histopathological analysis compatible with osteomyelitis of intro-operative bone samples, and at least two of the clinical criteria mentioned above (Centers for Disease Control and Prevention, 2016). Injuries of more than 3 months of evolution from onset or initial surgery were defined as chronic (Hotchen et al., 2017).

The data obtained for this initial sample were cross-checked against medical records and radiographic images kept at our institution to select the final cohort. This study was conducted with the approval of our institution's ethics committee.

We included patients aged ≥18 years old with a CPTO diagnosis of the femur or tibia treated with antibiotic cement-coated intramedullary nailing and a minimum follow-up of 24 months after nailing.

Exclusion criteria involved patients whose histopathological diagnosis of osteomyelitis (based on intraoperative samples) could not be confirmed on medical records, and those with non-union at the time of antibiotic nailing or segmental bone defects (SBDs) secondary to debridement or injury were excluded (Cierny–Mader type IV osteomyelitis) (Cierny et al., 2003).

### 2.1 Treatment protocol

Each case was analyzed separately. The surgery started with the removal of the previous osteosynthesis material. According to the preoperative plan, we made a bony window where the bone was most affected (e.g. sequestrum) and performed an aggressive surgical debridement, resecting sequestra, cloacae, and devitalized tissue until healthy bleeding (paprika sign) was reached (Tetsworth and Cierny, 1999; Patzakis and Zalavras, 2005; Simpson et al., 2001; Blanchette et al., 2018). The medullary cavity was progressively reamed and washed with 6 L of saline solution (Tetsworth and Cierny, 1999; Beals and Bryant, 2005). We took multiple (at least five) bone samples – including reamed material – for the histopathological analysis, culture, and antibiotic sensitivity test (Morgenstern et al., 2018; Chadayammuri et al., 2017; Hake et al., 2015). Afterwards, we implanted an antibiotic-coated nail 1.5–2 mm smaller in diameter than the last reamer used. When the last reamer was ≤13 mm, the metal core selected was an Ender nail (5 mm). When the last reamer was larger, an interlocking nail was implanted.

After surgery, patients were started on empirical systemic antibiotics (vancomycin 1 g/12 h; ceftazidime 2 g/8 h) until they were switched to specific ones according to culture results of intra-operative samples. According to the procedures of our institution, intraoperative samples were processed within 6 h. This process started with the homogenization of the tissues in brain–heart infusion (BHI) broth, and then they were inoculated on aerobic and anaerobic sheep blood agar, chocolate agar, and thioglycolate broth. Aerobic and anaerobic plates were incubated aerobically at 37 ${}^{\circ }$ C in 5 %–7 % CO ${}^{2}$ for 7 d and anaerobically at 37 ${}^{\circ }$ C for 14 d. Samples in thioglycolate were incubated for 14 d and sub-cultured on blood agar. Identification of germs that grew on the plates and antibiograms was performed with standard techniques.

Clinical and radiological controls were performed on weeks 3 and 6, on months 3, 6 and 12, and annually thereafter. Our institution's infectious disease department was in charge of antibiotic treatment and monitoring.

**Figure 1:**
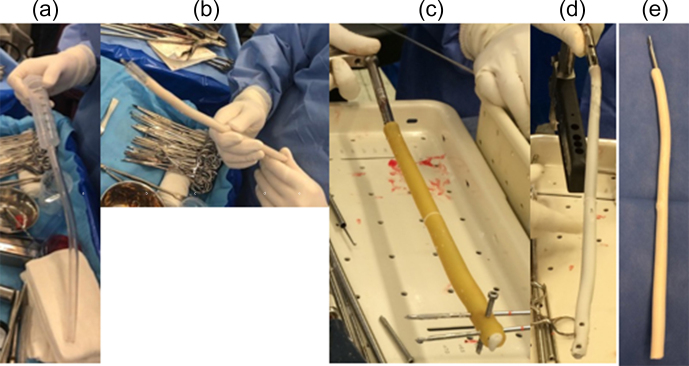
Images showing how ATB nails were manufactured in the operating room. **(a)** Preparation of the plastic chest tubes and the syringe for cement insertion. **(b)** Ender nail insertion into the cemented tube. **(c)** Insertion of the nail into the cemented tube and pines into the locking holes. **(d, e)** Images of the nail completion.

### 2.2 Nail coating technique

In order to design the metal core, plastic chest tubes of different diameters were used as molds based on preoperative planning and the diameter of the medullary canal. In all cases, the tube inner diameter was at least 2–3 mm larger than the diameter of the nail to be used. The tube was filled with antibiotic cement, and the nail was inserted. In the case of interlocking nails, a Steinmann pin was passed through the nail locking holes before adding the cement. The tube was allowed to set, sectioned lengthwise, and removed (Fig. 1). Physicians used Simplex cement (Stryker; Mahwah, NJ, USA) to manufacture the nails, adding vancomycin 2 g and gentamicin 0.5 g/40 g of cement.

Commercial nails (Subiton, Laboratorios SL, Buenos Aires, Argentina) had a stainless-steel core, interlocked, with 1 mm of gentamicin-impregnated cement (according to the manufacturer's specifications). According to medical records, surgeons used five nails with 665.0 mg of gentamicin, four with 698.1 mg, two with 732.6 mg, two with 642.4 mg, and one with 777.1 mg.

### 2.3 Definitions

Treatment was defined as successful when the infection was eradicated and the patient showed no clinical, laboratory, or radiographic signs or symptoms of infection for at least 24 months.

Additional procedure was defined as any reoperation performed after placement of the antibiotic-coated nail. Infection recurrence was defined as an initially controlled infection that relapsed and required new surgical treatment and nail exchange with or without systemic antimicrobial therapy. (Hake et al., 2015; Tice et al., 2003). Treatment failure was defined as the absence of infection control at the end of follow-up.

### 2.4 Variables analyzed

We analyzed patients' demographics, comorbidities, and relevant data about initial injury. Patients were classified using the Cierny–Mader classification based on comorbidities and osteomyelitis characteristics (Cierny et al., 2003).

We recorded all intraoperative complications related to the removal of the previous implant and the bone window, as well as the antibiotic used in the cement of custom-made nails manufactured in the operating room (Ender or interlocking nail) and in commercial nails, the soft tissue coverage procedures, operating time, and the results of intraoperative cultures.

Infection-related laboratory markers were also documented: erythrocyte sedimentation (ESR), normal value (nv) <16 mm/h; C-reactive protein (CRP), nv: <0.3 mg/dL, and white blood cell count (WBC), nv: <9000 /mm ${}^{3}$ , at the time of treatment and on the last control in patients' clinical records. The development, if any, of antibiotic-related allergies, nephrotoxicity, and ototoxicity was also recorded.

Infection control, reoperation, and failure rates at the end of follow-up were also registered.

### 2.5 Statistical analysis

Statistical analysis was performed using GraphPad PRISM-8.2 software, Inc., San Diego, USA. Quantitative data were expressed as median values and interquartile range (IQR), and non-continuous data were expressed as frequency and percentage. Fisher's nonparametric or Mann–Whitney tests were used to establish significant associations between variables. A value of p<0.05 was considered statistically significant.

## 3 Results

Out of the 55 patients identified in our database, 20 were excluded for presenting non-union or segmental bone defects, two for being under 18 years of age, two for not complying with minimum follow-up requirements, and one for lack of histopathological confirmation of chronic osteomyelitis in medical records.

**Table 1:** Summary description of the preoperative characteristics, treatment and evolution of the patients in the series.

Patients	Gender–	Bone injury	Prev. surgery	Cierny–	Treatment: previous	Soft tissue	Microbiology	Outcome	Follow-up
	age	Gustilo	( n )	Mader	implant/ATB nail type	coverage			(months)
1	M45	T 3B	4	3A	IMN/ILN	LMF	*P. mirabilis*	Failure	38
2	M56	T 2	2	3A	IMN/EN	–	MSSA	Control	45
3	M36	F	2	3A	IMN/EN	–	C-N	Control	92
4	M35	T	4	1A	IMN*A/EN	LMF	MSSA	Control	120
5	M22	F 3B	2	3A	EF/EN	VAC SG	MRSA	Control	121
6	M41	T	2	3A	IMN/EN	–	MSSA	Control	110
7	M70	F 3B	2	3B	IMN*A/ILN	–	CoNS	Control	50
8	M27	F 2	2	1A	IMN/ILN	–	MRSA	Control	32
9	M24	T 2	2	1A	IMN/EN	–	*E. faecalis*	Control	47
10	M18	F	3	3A	IMN/EN	–	MRSA	Control	42
11	M20	F	2	3A	IMN/ILN	–	MSSA	Control	40
12	M69	F 3B	9	1B	Plate/EN	–	P ${}^{2}$	Control	67
13	M47	F 2	2	1B	IMN/IN	VAC SG	MSSA	Control	68
14	F18	T 3C	3	3A	EF/IN	LMF	*P. aeruginosa*	Control	100
15	M29	T 3B	10	1A	IMN/IN	LMF	P ${}^{3}$	Control	100
16	F32	F 2	2	3A	IMN/IN	–	*E. faecalis*	Control	62
17	M33	T 3B	10	3A	IMN/IN	LMF	P ${}^{4}$	Control	89
18	M39	T 3B	3	3A	IMN*A/IN	LMF	*P. acnes*	Control	83
19	M53	F	1	1A	IMN/IN	–	MSSA	Control	52
20	M72	T	1	1B	IMN/IN	–	C-N	Control	67
21	M31	F	2	3A	IMN/IN	–	CoNS	Control	46
22	M41	F 3B	4	3A	IMN*A/IN	–	P ${}^{5}$	Control	29
23	M45	T 3A	3	3A	IMN/ILN	LMF	MRSA	Reop.	56
24	M29	F 3B	3	1A	IMN/ILN	–	MSSA	Reop.	65
25	M30	F 2	6	1A	IMN/EN	VAC SG	MRSA	Reop.	130
26	M24	T 3B	7	3A	IMN/EN	LMF	Poly ${}^{1}$	Reop.	79
27	M36	T 3B	8	1A	EF/IN	C-LEG	*P. aeruginosa*	Reop.	120
28	M35	F	7	3A	IMN/IN	–	MRSA	Reop.	55
29	M31	T	1	3A	Plate/IN	–	MRSA	Reop.	45
30	F22	T 2	4	3A	IMN/IN	LMF	P ${}^{6}$	Reop.	42

Gender: M: male; F: female. Injury: F: femur; T: tibia. Previous surgery: n : number. IMN: intramedullary nail; IMN A: antibiotic coated nail; EF: external fixation; EN: Ender nail; ILN: interlocking nail; IN: industrial nail; LMF: local muscle flap; VAC: vacuum-assisted closure; SG: skin graft; C-leg: cross-leg; C-N: culture-negative. MRSA: methicillin-resistent *Staphylococcus aureus*; MSSA: methicillin-sensitive *Staphylococcus aureus*; *P. aeruginosa*: *Pseudomona aeruginosa*; CoNS: Coagulase negative *Staphylococcus*; *E. Faecalis*: *Enterococcus faecalis*; *P. mirabilis*: *Proteus mirabilis*; *P. acnes*: *Propionibacterium acnes*; P ${}^{1}$ : MSSA + *Enterococcus cloacae*; P ${}^{2}$ : MRSA + *Proteus mirabilis* + *Klebsiella blee*; P ${}^{3}$ : *P. aeruginosa* + CoNS + MSSA; P ${}^{4}$ : CoNS + *Proteus mirabilis*; P ${}^{5}$ : *P. aeruginosa* + *Enterococcus cloacae*; P ${}^{6}$ : MRSA + *Klebsiella blee*. Reop.: reoperation.

### 3.1 Study population and patient characteristics

The final series was made up of 30 patients, 27 males and 3 females, with a median age of 33 (IQR 24–43) years. As regards pre-operative comorbidities, 4 patients were smokers, 2 were diabetic, and 1 presented chronic renal disease. None had a history of heart disease, anemia, poor vascularization (chronic lymphedema, venous stasis, major vessel compromise or arteritis), rheumatoid arthritis, immune deficiency, steroids or immunosuppressant medication, malnutrition, drug or alcohol abuse, or body mass index >30 . Twenty-eight patients were American Society of Anesthesiologists (ASA) I–II, and two were ASA III. Table 1 presents a description of patients.

The femur was the affected bone in 15 cases (9/15–60.00 % open fractures) and the tibia in 15 cases (11 open fractures, 73.3 %). All patients had previously undergone osteosynthesis, and the most frequent method used was intramedullary nailing (25; 83.33 %) – in four cases with antibiotic-coated nails. The median time between initial injury and antibiotic nail treatment was 10 (IQR 6.5–15) months. According to the Cierny–Mader classification, 18 (60.00 %) bone infections were classified as type 3A, 8 (26.66 %) as type 1A, 3 (10.00 %) as type 1B, and 1 (3.33 %) as 3B.

**Figure 2:**
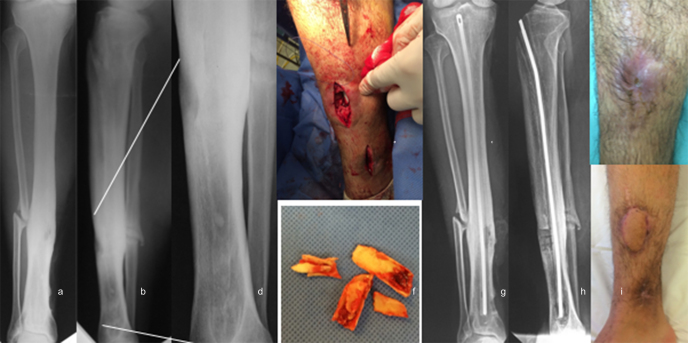
Osteomyelitis of the tibia treated with a custom-made ATB-cemented nail at another center. **(a–c)** AP and lateral radiographs showing intracanal cement debonding and clinical image with active fistula in the leg. **(d)** Higher zoom image of the lateral radiograph, to highlight the cement remnants inside the tibia. **(e, f)** Intraoperative images, showing the locations where the bone windows were made (at the level of the fistula and distally for the removal of the intracanal cement) and the cement remnants removed. **(g, h)** AP and lateral radiographs at 30 months after reaming and placement of an ATB-cemented nail with infection control. **(i)** Good evolution of the treatment of the soft tissue coverage defect.

Complications related to the removal of previous implants occurred in 75.00 % (three out of four) of the cases in which a custom-made antibiotic-coated nail was removed. In these cases, there was cement debonding inside the medullary canal, and a second bony window had to be opened for removal (Fig. 2). There were no complications related to the removal of non-coated intramedullary nails. We recorded no fractures associated with the bony window performed during debridement. Of the 30 patients, 13 (43.33 %) required soft-tissue coverage procedures. Nine of these procedures were performed on the leg (eight local muscle flaps and one cross-leg flap). Three thigh coverage defects were treated with negative pressure wound therapy and skin grafts.

### 3.2 Microbiological results

The mean number of samples sent for culture and histopathological analysis was five (range four–eight) Microbiological analysis of intraoperative samples revealed 28 (93.3 %) positive results, while all histopathological samples were positive. *Staphylococcus aureus* was the most frequently isolated germ ( 14/28 ; seven methicillin-resistant and seven methicillin-sensitive). Of the 14 staphylococcal infections, nine were controlled, and five required reoperations. One out of two *Pseudomona aeruginosa* infections and two out of six polymicrobial infections required reoperation. Failure occurred in a *Proteus mirabilis* infection. We found no significant differences in reoperations when infections were generated by an *S. aureus* vs. another microorganism ( p=0.99 ) or between mono- vs. polymicrobial infections ( p=0.64 ).

### 3.3 Laboratory outcomes

Humoral parameters showed a significant decrease in 29 cases at the end of the study. ESR decreased from 44.32±18.81 mm/h pre-operatively to 12.96±6.13 mm/h ( p=<0.0001 ) at the end of the study, CRP went from 9.15±9.96 to 0.3±0.29 mg/dL ( p=<0.001 ), and WBC went from 13518±3155 to 7400±2068 ( p=<0.0001 ). There were no cases of antibiotic allergy, nephrotoxicity, or ototoxicity.

### 3.4 Infection control rate

The overall median follow-up was 69 (range; 45–96) months. At the end of follow-up, 29 (96.6 %) patients were clinically well and showed no clinical, radiological, or laboratory evidence of infection. The infection control rate without the need for additional procedures was 70.00 % ( n=21 ).

### 3.5 Overall reoperation and failure rates

Eight (26.66 % – patients: 23 to 30) patients needed additional surgical debridement and replacement of the antibiotic-coated nail. In two of them, soft-tissue-covering procedures were also performed ( p : 23; 27). The median time to reoperation was 2.5 (range; 1–12) months. It should be noted that during nail removal, two of the custom-made antibiotic-coated nails showed nail–cement debonding. The overall rate for this complication was 62.50 % (five out of eight). In all cases the type of nail used for reoperation was the same as the one implanted in the first surgery. None of the patients who underwent these procedures required another surgery. When we comparatively analyzed the characteristics of reoperated and non-reoperated patients, we found no significant differences in terms of age ( p=0.18 ), sex ( p=0.99 ), affected bone ( p=0.68 ), open versus closed fractures ( p=0.52 ), number of previous surgeries ( p=0.18 ), time from the initial trauma to antibiotic-coated nail placement ( p=0.88 ), presence of soft tissue coverage defects ( p=0.42 ), use of commercial versus custom-made nails ( p=0.99 ), or microorganism isolated in intraoperative samples (SA vs. other ( p=0.99 ); mono- vs. polymicrobial p=0.64 ).

There was one failure (3.33 %) in a patient with a Gustilo 3B gunshot proximal tibial fracture referred to our center with an active fistula. The patient responded well to the initial treatment with an antibiotic-coated nail, but 11 months later his infection recurred. When the treatment was repeated, *S. aureus* was identified in addition to the infecting germ isolated during the first intervention (*Proteus mirabilis*). One month before the end of the study, (27 months after placement of the last antibiotic-coated nail) the infection recurred, and the patient is currently under treatment.

### 3.6 Commercial nails vs. custom nails

**Figure 3:**
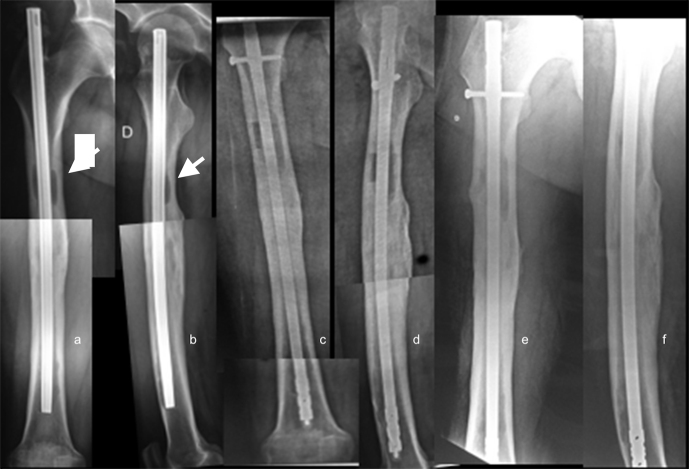
**(a, b)** AP and lateral radiographs of a patient with a history of open femur fracture treated with an endomedullary nail, 15 years of evolution. The patient had an active fistula at the level of the middle third of the external aspect of the thigh. Note the intracanal pathological images (white arrows). **(c, d)** AP and lateral radiographs 2 months postoperatively, after treatment with the commercial nail. **(e, f)** Control radiographs 48 months postoperatively with infection control.

Sixteen custom-made nails were used. In 10 of these cases, the metal core was an Ender nail, while the other 6 were treated with interlocking nails. The remaining 14 received commercial antibiotic-coated nails (Fig. 3).

In this series, the comparative analysis between custom-made and commercial nails showed no significant differences in preoperative variables (Table 2).

**Table 2:** Comparative analysis between patients treated with custom-made and commercial nails.

	Overall ( n 30)	Custom-made ( n 16)	Commercial nails ( n 14)	p<0.05
Gender (male) n (%)	27 (90)	16 (100)	11 (78.57)	0.09
Age median (IQR)	33 (24–43)	32.5 (24–45)	34 (30.5–42.5)	0.7
Risk factors n (%)				
Diabetes	2 (6.66)	1 (6.25)	1 (7.14)	0.9
Smoking	4 (13.33)	3 (18.75)	1 (7.14)	0.6
CRD	1	1	–	0.9
ASA I–II	28 (93.33)	15 (93.75)	13 (92.85)	0.9
ASA III–IV	2 (6.66)	1 (6.25)	1 (7.14)	
Bone – open fracture n				
Femur	15–9	9–6	6–3	0.3
Tibia	15–11	7–5	9–6	0.3
Number of previous surgery median (range)	3 (1–10)	2.5 (2–9)	3 (1–10)	0.7
Previous osteosynthesis type n (%)	30	16	14	–
IMN	25 (83.33)	14–2* (87.5)	11–2* (78.57)	
EF	3 (10)	1 (6.25)	2 (14.28)	
Plate	2 (6.66)	1 (6.25)	1 (7.14)	
Months from injury to ATB nail median (IQR)	10 (6.5–15)	9.5 (6.5–12)	12 (6–20.7)	0.18
Cierny–Mader n (%)				
1A	8 (26.66)	5 (31.25)	3 (21.43)	0.4
3A	18 (60.00)	9 (56.25)	9 (64.28)	0.7
1B	3 (10.00)	1 (6.25)	2 (14.28)	0.6
3B	1 (3.33)	1 (6.25)	–	0.9
ATB nail (metal core) n (%)				
Ender	10 (33.33)	10 (62.50)	–	
Locking nail	20 (66.66)	6 (37.50)	14 (100)	
ATB in PMMA n (%)				
Vancomycin + gentamicin	16 (53.33)	16 (100)		
Gentamicin	14 (46.66)	14 (100)		
Soft tissue coverage	13 (43.33)	6 (37.50)	7 (50.00)	0.7
Surgical time median (IQR)	123	130.5	100.5	<0.001
	(110.3–131.8)	(122.8–138)	(94.2–121)	
Intraoperative culture n (%)				
MRSA	7 (23.33)	5 (31.25)	2 (14.28)	0.4
MSSA	7 (23.33)	5 (31.25)	2 (14.28)	0.4
Polymicrobial	6 (20.00)	2 (12.50)	4 (28.57)	0.3
Culture-negative		P ${}^{1-2}$	P ${}^{3-6}$	
	2 (6.66)	1 (6.25)	1 (7.15)	0.9
Others	8 (26.66)	3 (18.75)	5 (37.71)	0.4
Infection control n (%)	29 (96.60)	15 (93.7)	14 (100)	0.9
Reoperation n (%)	8 (26.66)	4 (25.00)	4 (28.57)	0.9
Failure n (%)	1 (3.33)	1 (6.25)	–	0.9
Follow-up months, median (IQR)	65	60.5	64.5	0.8
	(45–96)	(42.7–105.5)	(44.2–91.7)	

CRD: chronic renal disease. *: ATB nail. IMN: intramedullary nail. EF: external fixation. Gent: gentamicin. Van: vancomycin. MRSA: methicillin-resistent *Staphylococcus aureus*. MSSA: methicillin-sensitive *Staphylococcus aureus*. P ${}^{1}$ : MSSA + *Enterococcus cloacae*. P ${}^{2}$ : MRSA + *Proteus mirabilis* + *Klebsiella blee*. P ${}^{3}$ : *P. aeruginosa* + CoNS + MSSA. P ${}^{4}$ : CoNS + *Proteus mirabilis*. P ${}^{5}$ : *P. aeruginosa* + *Enterococcus cloacae*. P ${}^{6}$ : MRSA + *Klebsiella blee*. The p value corresponds to the comparative analysis between custom-made and industrial nails.

We found no significant differences between nail types in terms of infection control ( p=0.99 ), reoperation rates ( p=0.99 ), or failure rates ( p=0.99 ).

## 4 Discussion

The main study finding is that infection control in CPTO was achieved in 96.66 % of the patients using antibiotic cement-coated nails.

The use of antibiotic-loaded cement or PMMA has proven effective to administer local antimicrobials, fill voids, and achieve >90 % healing rates in infected joint replacements (Hanseen, 2005). Antibiotic-coated nails present an alternative to extend this concept and represent a successful therapeutic option widely supported by the literature (Beals and Bryant, 2005; Qiang et al., 2007; Thonse and Conway, 2007).

Several manufacturing techniques have been described for these devices, like using pins, rods, and flexible or locking nails (Patzakis and Zalavras, 2005; Gosselin et al., 2004; Paley and Herzenberg, 2002). These techniques offer the advantage of delivering high doses of antibiotics locally, with few or no systemic adverse effects (Zalavras et al., 2004). The peak of antibiotic elution is reached 24 h after placement and remains stable until day 10. This elution is maintained at high doses until week 10 and may persist in lower concentrations for up to 36 weeks (Nelson et al., 1994). In addition, interlocking nailing also provides mechanical stability, if needed (Thonse and Conway, 2007).

Conway et al. (2014) published a series of 43 patients with infected long-bone non-unions (Cierny–Mader type IV) treated with custom-made antibiotic-coated nails. They reported 100 % consolidation and infection control with a reoperation rate of 40 %, mostly related to SBDs. The fact that we excluded type IV osteomyelitis – which requires a greater number of surgeries for bone reconstruction – may account for the difference in reoperation rates.

Jorge-Mora et al. (2019) analyzed a series of 10 PTO patients treated with commercially available antibiotic-coated nails (the same used for our series). They reported infection control in all cases, with only one reoperation. Even though we observed no failures with this type of antibiotic-coated nail, we found a higher reoperation rate.

Shyam et al. (2009) reported on 25 patients with infected non-union of the femur and tibia treated with antibiotic-coated Ender nails. They described 100 % infection control and a need for subsequent nail replacement in 10 (40 %) patients. In our series, we used 10 Ender nails in consolidated fractures and observed a 20 % (2 out of 10) need for additional procedures because of lack of infection control.

In more than 83 % of the cases in our series, CPTO occurred after nailing. In these cases, infection control results were similar to those described by Qiang et al., 2007), who reported a success rate of 94.7 % (19 patients) using antibiotic cement-coated pins. These authors recommend the use of external fixation in case of non-union with this type of metal core. We agree with these authors that the use of an Ender nail or a non-locking nail in cases of infected long bone non-union or SBDs requires another device to provide stability, which represents a limitation in this indication.

Another treatment option with recently reported good results for medullar cavity reaming and lavage is the combined use of the reamer–irrigator–aspirator (RIA) system and antibiotic-coated nails (Kanakaris et al., 2014; Finelli et al., 2019). By using the RIA system, surgeons do not need to create a bone window, and, therefore, the procedure morbidity is reduced. Still, we found no such complication in our series.

Eight (26.66 %) patients in our series required a new intervention to control an infection. Reoperations were more frequent (though not significantly) when the affected bone was the tibia ( n=5 ; 62.50 %), the original injury was an open fracture ( n=6 ; 75.00 %), soft tissue defects were present ( n=5 ; 62.50 %), and the isolated germ was *S. aureus* ( n=5 ; 62.50 %). Our reoperation rate was similar or even lower than the 20 %–40 % reported by previous series (Shyam et al., 2009; Garcia et al., 2018).

We understand that surgical debridement is essential to treat osteomyelitis successfully. Therefore, insufficient debridement could account for our reoperation rates. Simpson et al. (2001) demonstrated that a vital bone resection margin reduces the possibility of recurrence. Although we found no significant differences between reoperated and non-reoperated patients in terms of isolated microorganism, this could have an effect on reoperation rates. In our analysis, 50 % (1 out of 2) *P. aeruginosa* and 35.71 % (5 out of 14) *S. aureus* infections recurred, which is consistent with the findings of Tice et al. (2003), who reported *P. aeruginosa* infections are associated with a higher risk of recurrence than *S. aureus*. On the other hand, Jorge et al. (2018) reported that polymicrobial PTO is associated with worse outcomes. In our series, 33.33 % (2 out of 6) polymicrobial infections needed reoperation.

The population analyzed in this series and treated either with custom-made or commercial antibiotic-coated nails obtained similar results, and we observed no significant differences in their preoperative characteristics. In our analysis, devices manufactured in the OR were associated with longer operating times and some difficulties at the time of nail removal due to nail–cement debonding. While other authors have reported a 20 % incidence of this complication (Wasko and Borens, 2013; Thonse and Conway, 2007; Shyam et al., 2009), it was more than 3 times higher in our series, as fragment extraction added to the complexity of the procedure and the need for an extra bone window. On the other hand, commercially available nails were easier to remove and did not show nail–cement debonding, possibly due to the greater strength of the cement used in their manufacturing process. Finally, we found no significant differences between the two types of nails in terms of infection control and need for reoperation.

The limitations of this study are those inherent to retrospective studies with a small sample. This might account for the lack of statistical significance of some of the variables analyzed producing a type 2 error. This number was limited as we excluded Cierny–Mader type IV osteomyelitis cases to obtain a more homogeneous population – especially with regard to procedures related to SBD reconstruction. Another limitation is the lack of a control group or functional analysis. On the other hand, the strengths of this study include the detailed characterization of patients with a complex and difficult to treat condition such as CPTO using antibiotic-coated nails. The literature does not include many comparative studies on the results of patients treated with commercial nails versus those treated with custom-made nails. Finally, our study presents an acceptable follow-up (minimum of 24 months) considering most recurrences occur during the first postoperative year.

## 5 Conclusion

Along with the classic treatment pillars for chronic osteomyelitis, the use of antibiotic cement-coated nails proved useful in the challenging treatment of post-traumatic chronic osteomyelitis. Our comparative analysis of custom-made and commercial antibiotic-coated nails did not show significant differences in terms of infection control and reoperation rates for patients without non-union or segmental bone defects. However, the use of custom-made nails made in the operating room was associated with longer operating times and nail–cement debonding at the time of extraction. In order to determine the external validity of this study, it is necessary to conduct properly designed studies in a larger population.

## Data Availability

All data generated and analyzed during this study are included in this published article and are available from the corresponding author on reasonable request.
